# Quadruple perovskite ruthenate as a highly efficient catalyst for acidic water oxidation

**DOI:** 10.1038/s41467-019-11789-3

**Published:** 2019-08-23

**Authors:** Xianbing Miao, Lifu Zhang, Liang Wu, Zhenpeng Hu, Lei Shi, Shiming Zhou

**Affiliations:** 1 Hefei National Laboratory for Physics Sciences at the Microscale, University of Science and Technology of China, Hefei, Anhui 230026 P. R. China; 2 School of Physics, Nankai University, Tianjin, 300071 P. R. China

**Keywords:** Catalyst synthesis, Electrocatalysis, Materials for energy and catalysis

## Abstract

Development of highly active and durable oxygen-evolving catalysts in acid media is a major challenge to design proton exchange membrane water electrolysis for producing hydrogen. Here, we report a quadruple perovskite oxide CaCu_3_Ru_4_O_12_ as a superior catalyst for acidic water oxidation. This complex oxide exhibits an ultrasmall overpotential of 171 mV at 10 mA cm^−2^_geo_, which is much lower than that of the state-of-the-art RuO_2_. Moreover, compared to RuO_2_, CaCu_3_Ru_4_O_12_ shows a significant increase in mass activity by more than two orders of magnitude and much better stability. Density functional theory calculations reveal that the quadruple perovskite catalyst has a lower Ru 4d-band center relative to RuO_2_, which effectively optimizes the binding energy of oxygen intermediates and thereby enhances the catalytic activity.

## Abstract (web summary)

Electrocatalytic water splitting provides a renewable path to store energy in chemical bonds, but highly efficient oxygen-evolving catalysts in acid media remain limited. Here the authors report a quadruple perovskite ruthenate oxide as an effective and stable oxygen evolution electrocatalyst.

## Introduction

Proton exchange membrane water electrolysis (PEMWE) has sparked widely attention as a most promising technology for direct conversion of electrical energy into fuels by electrochemical water splitting^1–3^. Essential to the water electrolyzers is oxygen evolution reaction (OER) taking place at anode, where a complex multistep proton-coupled electron transfer process generates a sluggish kinetics^3–5^. In acid PEMWE, precious-metal oxides of IrO_2_ and RuO_2_ are currently regarded as the most active OER catalysts^6^. However, high costs and still poor intrinsic activities greatly limit their catalytic efficiencies. Therefore, developing efficient catalysts with less content of precious-metal and higher intrinsic activity is quite appealing for acidic water oxidation.

Recently, the synthesis of complex oxides has been reported to be a promising approach for both reducing the precious-metal usage and promoting the intrinsic activity^7–13^. Various complex oxides with less precious-metal contents, including perovskite^7–10^ and pyrochlore-type^11–13^ compounds, were found to exhibit higher intrinsic OER activities relative to the simple binary oxides under acid conditions. For instance, the specific activity reached to about 2.8 mA cm^−2^_oxide_ at 1.55 V for perovskite oxide Ba_2_NdIrO_6_, which is about 14 times larger than that of the commercial IrO_2_ (∼0.2 mA cm^−2^_oxide_)^8^. A more efficient IrO_*x*_/SrIrO_3_ catalyst with a high specific current density of 10 mA cm^−2^_oxide_ at 1.50 V was revealed by strontium leaching from surface layers of SrIrO_3_ thin films during electrochemical testing^9^. Similar promotions were also observed in pyrochlore-type Y_2_Ir_2_O_7_ and Y_2_Ru_2_O_7-δ_ catalysts^12,13^. Compared to iridium, ruthenium is much lower in price^4,6,13^. Moreover, ruthenium oxide typically exhibits higher activity than iridium oxide^4,6^. Therefore, the ruthenium oxides with complex structures represent promising catalysts for acidic water oxidation.

In this work, we report a complex perovskite oxide CaCu_3_Ru_4_O_12_ as a highly efficient catalyst for OER in acid. Unexpectedly, this ruthenate catalyst presents an ultralow overpotential of 171 mV at 10 mA cm^−2^_geo_ in 0.5 M H_2_SO_4_ solution, surpassing the most reported robust OER catalysts in acid up to date. Moreover, it achieves large mass activity of 1942 A g^−1^_Ru_ and specific activity of 22.1 mA cm^−2^_oxide_ at 1.50 V, which are 170 and 96 times higher than those of the commercial RuO_2_, respectively. The long-term durability tests also reveal that CaCu_3_Ru_4_O_12_ shows much better stability than RuO_2_. The excellent OER performance of this ruthenate oxide would make it a promising catalyst in commercial PEMWE.

## Results

### Synthesis and characterization

**Fig. 1:**
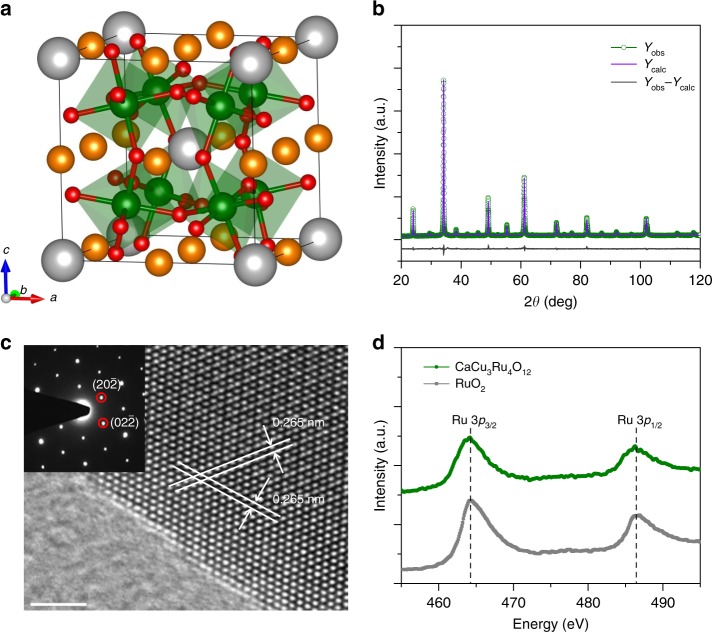
Structural characterization of CaCu_3_Ru_4_O_12_. **a** Crystal structure of CaCu_3_Ru_4_O_12_. Color code: Ca (gray), Cu (bronze), Ru (green), and O (red). **b** XRD pattern for CaCu_3_Ru_4_O_12_ together with the Rietveld refined results. **c** HRTEM image and SAED pattern (inset) for CaCu_3_Ru_4_O_12_. The scale bar is 2 nm. **d** Ru 3p XPS spectra

The ruthenate oxide CaCu_3_Ru_4_O_12_ belongs to the typical A-site ordered quadruple perovskite compound AA'_3_B_4_O_12_, whose crystal structure can be considered as a 2 × 2 × 2 superstructure of simple cubic perovskite ABO_3_. As shown in Fig. 1a, one Ca^2+^ and three Cu^2+^ ions occupy A-sites in order, while the Ru^4+^ ions form corner-share RuO_6_ octahedra at B-sites^14,15^. The polycrystalline powders of this compound were synthesized by a conventional solid-state reaction. Scanning electron microscopy (SEM) image displays that the particle sizes are submicron (Supplementary Fig. 1). Figure 1b shows the powder X-ray diffraction (XRD) of as-prepared sample, where all the diffraction lines are well indexed to the cubic (*Im-3*) phase without detectable amount of impurities. On the base of Rietveld refinements on diffraction data, the lattice parameter *a* and Ru–O bond length are determined to be 7.4206(3) and 1.9808(7) Å (Supplementary Table 1), respectively, which well agrees with the reported values^14^. The good crystallinity and single-phase structure are further confirmed by high resolution transmission electron microscopy (HRTEM) image together with selected area electron diffraction (SAED) pattern (Fig. 1c). The observed lattice fringes with inter-plane spacing of 0.265 nm correspond to the $\left( {02\bar 2} \right)$ or $\left( {20\bar 2} \right)$ plane of cubic phase CaCu_3_Ru_4_O_12_. X-ray photoelectron spectra (XPS) measurements (Fig. 1d) reveal that CaCu_3_Ru_4_O_12_ exhibits two peaks at 464.2 and 486.3 eV, which are similar to those of RuO_2_ and assigned to 3p_3/2_ and 3p_1/2_ states of Ru^4+^ ions^16^, respectively. The energy dispersive X-ray spectrum (EDS, Supplementary Fig. 2) demonstrates that the molar ratio of Ca/Cu/Ru is 1:2.98:4.03, which suggests a good stoichiometry of the as-synthesized sample.

### OER performance

**Fig. 2:**
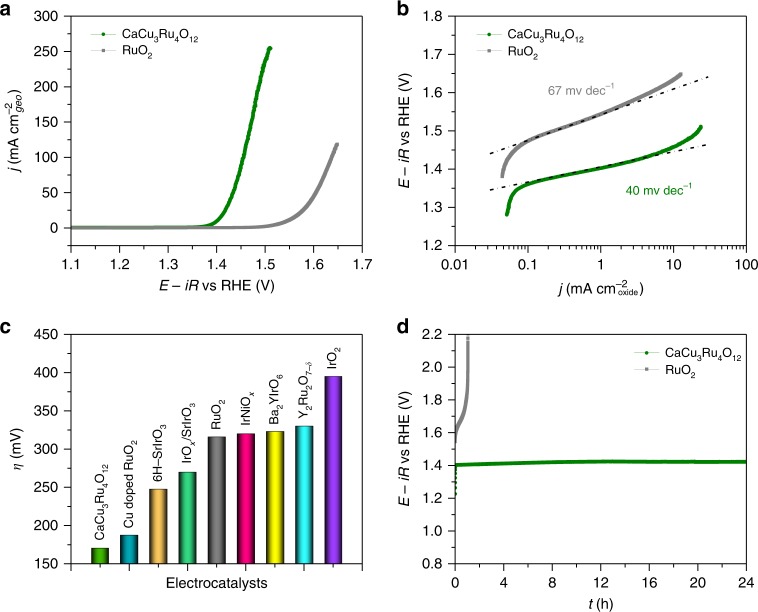
Electrochemical OER performance. **a** Polarization curves of CaCu_3_Ru_4_O_12_ and the commercial RuO_2_ measured in O_2_-saturated 0.5 M H_2_SO_4_ solution. **b** Tafel plots of specific OER activity. **c** Comparison of the overpotentials at 10 mA cm^−2^_geo_ for CaCu_3_Ru_4_O_12_ and recent reported OER catalysts in acid media. **d** Chronopotentiometric measurements of CaCu_3_Ru_4_O_12_ and the commercial RuO_2_ at 10 mA cm^−2^_geo_

We evaluated the electrocatalytic OER activity of CaCu_3_Ru_4_O_12_ in O_2_-saturated 0.5 M H_2_SO_4_ solution using a standard three-electrode system. Figure 2a shows the polarization curve for CaCu_3_Ru_4_O_12_, along with the commercial RuO_2_ as a reference. For RuO_2_, a large overpotential (*η*) of 316 mV was required to achieve a current density of 10 mA cm^−2^_geo_, which is in line with those previous reports (Supplementary Table 2). Intriguingly, for CaCu_3_Ru_4_O_12_, the current density is remarkably enhanced and the overpotential is much reduced to 171 mV. Moreover, by normalizing the current density to catalyst mass or Ru metal mass, a significant increase in the mass activity is observed (Supplementary Fig. 3). For example, the mass activity of CaCu_3_Ru_4_O_12_ achieves 1942 A g^−1^_Ru_ at 1.50 V, which is 170 times higher than that of RuO_2_ (11.4 A g^−1^_Ru_). As shown by Tafel plots in Fig. 2b, this enhanced performance is further verified to be intrinsic by the specific activity, which is obtained from normalizing the mass activity with Brunauer-Emmett-Teller (BET) surface area (Supplementary Fig. 4). Notably, the specific current density of CaCu_3_Ru_4_O_12_ at 1.50 V reaches up to the value of 22.1 mA cm^−2^_oxide_, which is 96 times larger than that of RuO_2_ (0.23 mA cm^−2^_oxide_). The similar feature is also found in the specific activities normalized by the electrochemically active surface areas (ECSA), which are derived from the electrochemical double-layer capacitance measurements (Supplementary Fig. 5, 6). Meanwhile, the Tafel plots illustrate that CaCu_3_Ru_4_O_12_ presents a smaller Tafel slope of 40 mV dec^−1^ than RuO_2_ of 67 mV dec^−1^, suggesting its significantly accelerated OER kinetic. The faster OER kinetic rate is further reflected by the electrochemical impedance spectroscopy (EIS) measurements, where a remarkable decrease of charge transfer resistance (Rct) is revealed for the ruthenate catalyst (Supplementary Fig. 7).

Our electrochemical tests clearly demonstrate that CaCu_3_Ru_4_O_12_ exhibits a superior activity for acidic water oxidation. To the best of our knowledge, this complex oxide has the lowest overpotential at 10 mA cm^−2^_geo_ among the excellent OER catalysts in acid electrolytes as reported up to date (Fig. 2c and Supplementary Table 3). Furthermore, its intrinsic activity also outperforms all of them. Particularly, the specific current density of the ruthenate at 1.50 V is even about two-fold higher than that of the recently reported best oxide catalyst IrO_*x*_/SrIrO_3_ (Supplementary Table 3).

To access the electrochemical stability of CaCu_3_Ru_4_O_12_, we performed a long-term chronopotentiometry at a constant current density of 10 mA cm^−2^_geo_, together with RuO_2_ for comparison. As shown in Fig. 2d, the commercial RuO_2_ nearly loses all its activity after 1 h. In constrast, the overpotential for CaCu_3_Ru_4_O_12_ just slightly increases from 171 to 192 mV after a 24 h continuous operation. The high stability of this complex oxide is also supported by the XRD, XPS, and HRTEM measurements after the OER test. No visible changes in the positions of XRD diffraction lines and XPS core level peaks (Supplementary Fig. 8) are found on the materials before and after the OER testing, while there is a marked decrease in the intensities due to the less catalyst mass used in the measurements after the OER testing. HRTEM image (Supplementary Fig. 9) further shows that the lattice fringes for the CaCu_3_Ru_4_O_12_ particle after the durability test still extend all the way to the surface, which suggests that the surface structure of CaCu_3_Ru_4_O_12_ is maintained during OER. In addition, inductively coupled plasma atomic emission spectroscopy (ICP-AES) measurements (Supplementary Table 4) reveal that less than 6.7% Ca, 2.9% Cu, and 2.7% Ru ions are found to be dissolved in the electrolyte, indicating just a slight cation leaching during the OER operation. This is also confirmed by TEM-EDS spectrum (Supplementary Fig. 10), which shows a small change of Ca/Cu/Ru ratio for ruthenate catalyst after the acidic OER testing. These results suggest that this ruthenate oxygen-evolving catalyst shows good chemical stability in acid, which will be beneficial for the commercial applications.

## Discussion

**Fig. 3:**
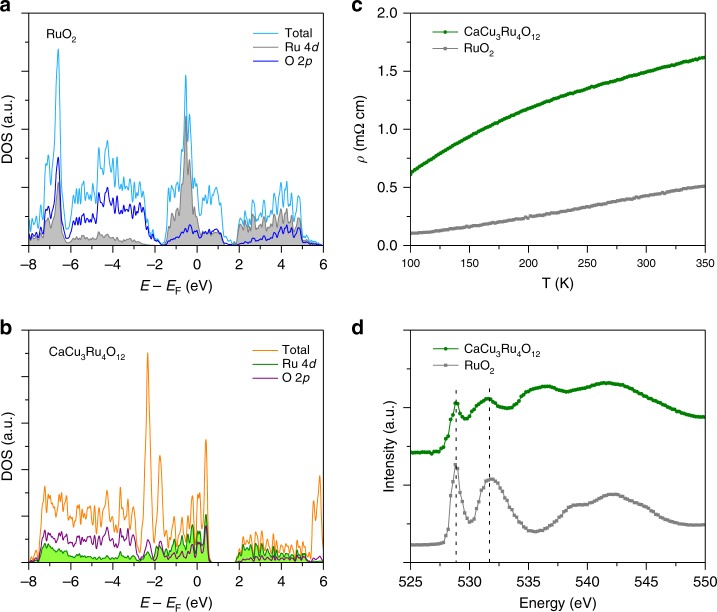
Electronic structure studies. **a, b** Computed density of states (DOS) of **a** RuO_2_ and **b** CaCu_3_Ru_4_O_12_. **c** Temperature dependence of resistivity. **d** O *K*-edge XAS

To understand the enhanced OER activity in the complex oxide CaCu_3_Ru_4_O_12_, density functional theory (DFT) calculations were carried out. Figures 3a, b show the computed density of states (DOS) for RuO_2_ and CaCu_3_Ru_4_O_12_, respectively. The large DOS from Ru 4d and O 2p bands cross the Fermi level for both the oxides, which indicates that they present an intrinsically metallic behavior. However, compared to RuO_2_, CaCu_3_Ru_4_O_12_ exhibits a downshift in the projected DOS for both Ru 4d band and O 2p band relative to the Fermi level. For RuO_2_, the calculated Ru 4d band and O 2p band centers are 0.72 and 0.16 eV, while the values are reduced to −1.37 and −3.42 eV for CaCu_3_Ru_4_O_12_, respectively. To confirm these electronic features, we further conducted the electrical transport measurements. Figure 3c plots the temperature-dependent resistivity for RuO_2_ and CaCu_3_Ru_4_O_12_. Upon cooling, their electrical resistivities show a monotonous decrease. This temperature dependence reveals an intrinsic metallic state for both the oxides, agreeing well with the DFT calculations. The metallic conductivity could ensure fast charge transfer between catalyst-electrolyte and catalyst-support electrode interfaces, which is beneficial for the OER process^17^. Moreover, the resistivity of CaCu_3_Ru_4_O_12_ is apparently higher than that of RuO_2_ within the measured temperature range. For example, CaCu_3_Ru_4_O_12_ exhibits a more than threefold increase in the resistivity (1.5 mΩ cm) at room temperature compared to RuO_2_ (0.43 mΩ cm). The larger resistivity is supposed to be associated with the Ru 4d and O 2p band centers far away from the Fermi level owing to their downshifts to low energies.

During the OER process, the conventional adsorbate mechanism demonstrates that the electrochemical activity of catalysts is determined by the binding strengths of adsorbed intermediates such as HO*, O*, and HOO* to active sites^18–22^. For transition metal oxide OER catalysts, the d-band center of active metal sites is closely related to the binding strength between metal sites and adsorbed oxygen species^22–24^. Usually, a lower d-band center generates a weaker metal-oxygen binding^22–24^. In our case, the refined Ru–O bond length of CaCu_3_Ru_4_O_12_ is 1.9808(7) Å, which is longer than the average Ru–O bond length of RuO_2_ (~1.97 Å)^25,26^. This implies that CaCu_3_Ru_4_O_12_ has a weaker Ru–O bonding strength compared to RuO_2_, well associated with that the former has a lower Ru 4d-band center. To further assess the strength of Ru–O bonds in our catalysts, we performed the O K-edge X-ray absorption spectroscopy (XAS). O 1 s spectra in K-edge XAS reflect the transition from O 1 s core level to unoccupied O 2p states hybridized with metal ions^27,28^. A higher absorption intensity of O 1 s spectra suggests a stronger hybridization between oxygen and metal ions. As shown in Fig. 3d, two pre-edge peaks are clearly observed at 528.8 and 531.7 eV for our catalysts, which are assigned to the unoccupied orbitals of O 2p hybridized with Ru 4d t_2g_ and e_g_ orbitals^29,30^, respectively. The intensities of pre-edge peaks normalized by the adsorption background at the energy ranges both below the absorption edge and at ∼550 eV^27,28,31^ are used to estimate the hybridization of O 2p with Ru 4d orbitals. Obviously, the normalized intensities for CaCu_3_Ru_4_O_12_ are much smaller than those for RuO_2_, which implies that the Ru–O bonds in the complex oxide have a less hybridization. This feature further supports that a weaker Ru–O binding strength occurs in CaCu_3_Ru_4_O_12_ owing to its lower Ru 4d band center.

**Fig. 4:**
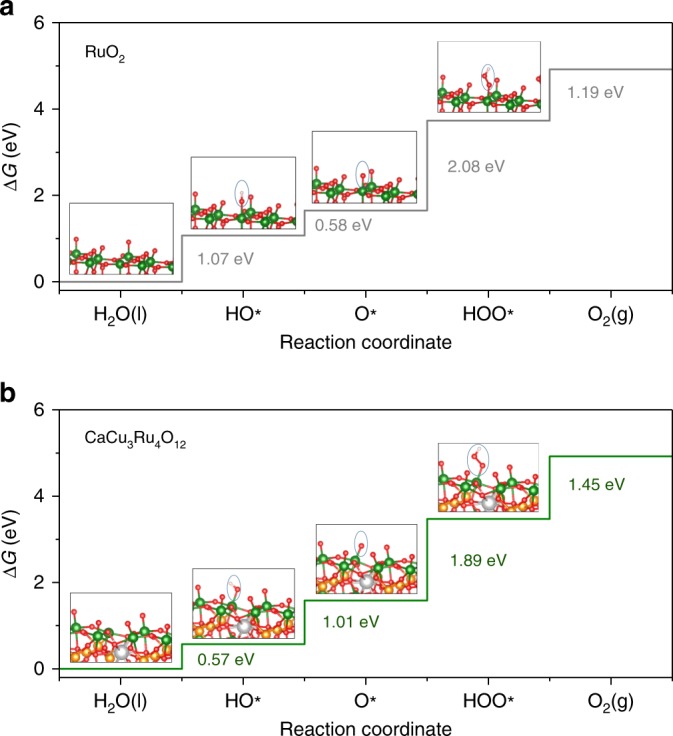
Calculated free energy diagrams. **a** RuO_2_. **b** CaCu_3_Ru_4_O_12_. The optimized structures of HO*, O*, and HOO* adsorptions on the surfaces are shown in the insets

To elucidate the role of the electronic structures on OER energetics for our Ru-based oxides, theoretical OER overpotentials were further evaluated from DFT calculations. According to the previous studies^18–22^, we assumed a four-step OER mechanism, which proceeds through four consecutive proton and electron transfer steps with HO*, O*, and HOO* intermediates. The Gibbs free energies of different intermediates adsorbed by surface Ru atoms for both catalysts were calculated with the standard hydrogen electrode (SHE) method^32,33^. Firstly, we focused on RuO_2_ (110) and CaCu_3_Ru_4_O_12_ (001) surfaces (Supplementary Fig. 11, 12), since they are considered as the relatively more stable facets for rutile and pervoskite oxides^16,19,20^, respectively. Figure 4a, b illustrates the free energy diagrams calculated on both the surfaces. For RuO_2_, all reaction steps move uphill in free energy at zero potential. Moreover, the free energy difference between Δ*G*_HOO*_ and Δ*G*_O*_ is found to be maximum, i.e., Δ*G*_HOO*_ − Δ*G*_O*_ = 2.08 eV, indicating that a minimum potential of *U* = 2.08 V has to be applied to make every step downhill in free energy. This means that the formation of HOO* from O* intermediates is the rate-determining step (RDS), which results in a high OER overpotential of 0.85 V. Our calculations on RuO_2_ are in accord with previous reports^16,34,35^. For CaCu_3_Ru_4_O_12_, the formation of HOO* intermediates is still the RDS. However, Δ*G*_HOO*_ − Δ*G*_O*_ is significantly reduced to 1.89 eV, corresponding to a theoretical overpotential of 0.66 V. These calculated results imply that compared to RuO_2_ the quadruple perovskite ruthenate exhibits a much reduction in the OER overpotential by 0.19 V. Notably, our calculated OER overpotentials are different from the experimental values possibly because the formers are just obtained from the thermodynamic analyses^10^. However, the magnitude of reduction in the overpotentials is comparable between them (0.19 V vs 0.145 V), which supports that the unique electronic structure associated with the quadruple perovskite crystal structure for CaCu_3_Ru_4_O_12_ would be responsible for the observed superior OER activity.

Additionally, we also carried out the DFT calculations on other facets for both the catalysts. For RuO_2_, as shown in Supplementary Fig. 13, 14, the free energy diagrams for less stable (001) and (100) surfaces illustrate that the theoretical overpotentials are 1.00 and 0.74 V, respectively, which are still larger than that of CaCu_3_Ru_4_O_12_ (001) surface. For CaCu_3_Ru_4_O_12_, the calculations on (110) surface (Supplementary Fig. 15) reveal that this high-index facet exhibits an inferior OER activity with a theoretical overpotential of 0.85 V. Furthermore, in order to evaluate the role of Ca and Cu cations on the OER activity, we calculated the free energy diagrams on Ca and Cu sites for CaCu_3_Ru_4_O_12_ (001) surface, as shown in Supplementary Fig. 16, 17. The theoretical overpotentials are estimated to be 0.99 and 0.92 V, respectively, which both are much larger than that on Ru sites (0.66 V). This result strongly supports that Ru cations are the OER active sites for CaCu_3_Ru_4_O_12_.

Recent theoretical studies on the OER mechanism for transition metal oxides have demonstrated that the Δ*G*_O*_ − Δ*G*_HO*_ difference can effectively describe their OER activities since there is a strong relations between Δ*G*_HO*_ and Δ*G*_HOO*_^19–22^. Actually, this difference reflects the metal-oxygen binding strength^22^. The optimal catalysts are required to have neither too strong nor too weak binding strengths. Too strong metal-oxygen bond strength would result in a small Δ*G*_O*_ − Δ*G*_HO*_ difference and then a large Δ*G*_HOO*_ − Δ*G*_O*_ difference. Our DFT calculations reveal CaCu_3_Ru_4_O_12_ exhibits a much reduction in Δ*G*_HOO*_ − Δ*G*_O*_ difference with comparison to RuO_2_. Moreover, this reduction is accompanied by a large increase in Δ*G*_O*_ − Δ*G*_HO*_ from 0.58 to 1.01 eV. In other words, the weaker Ru–O coupling in CaCu_3_Ru_4_O_12_ leads to a larger Δ*G*_O*_ − Δ*G*_HO*_ and then a smaller Δ*G*_HOO*_ − Δ*G*_O*_, i.e., a smaller overpotential. Therefore, we conclude that the weaker binding strength between oxygen species and Ru sites due to the lower Ru 4d band center gives rise to the higher intrinsic activity of quadruple perovskite CaCu_3_Ru_4_O_12_. In addition, previous studies revealed that the instability for RuO_2_ may be attributed to the high Ru dissolution rate due to oxygen vacancies arising from the participation of activated lattice oxygen in OER process^36^. For transition metal oxides, the strong metal-oxygen couplings were reported to easily trigger the lattice oxygen redox during OER^37^. We thus suppose that the weaker Ru–O binding strength in CaCu_3_Ru_4_O_12_ may also lower the Ru dissolution rate owing to the less participation of lattice oxygen redox in the OER process, which will improve its stability in acid.

In summary, we develop a complex ruthenate CaCu_3_Ru_4_O_12_ as an efficient OER catalyst with excellent activity and good durability in acidic media. It only requires an ultralow overpotential of 171 mV to yield a current density of 10 mA cm^−2^_geo_ under pH = 0. The mass activity reaches up to 1942 A g^−1^_Ru_ at 1.50 V, which is 170 times higher than that of the state-of-the-art RuO_2_. Moreover, this complex oxide exhibits much better stability than RuO_2_. Electronic structure studies and DFT calculations reveal that compared to that of RuO_2_, the adsorption of oxygen intermediates for CaCu_3_Ru_4_O_12_ is significantly weakened due to its lower Ru 4d-band center, which facilitates the sluggish kinetics of oxygen-evolving process and enhances the intrinsic activity. This work not only demonstrates that the quadruple perovskite ruthenate holds great potential in the commercial application of PEMWE, but also provides a guidance for exploiting superior oxygen-evolving catalysts from the materials with complex structures.

## Methods

### Synthesis and characterizations

The polycrystalline powders of quadruple perovskite oxide CaCu_3_Ru_4_O_12_ were prepared by a conventional solid-state reaction. Stoichiometric mixtures of 0.2002 g CaCO_3_, 0.4773 g CuO, and 1.0645 g RuO_2_ were thoroughly ground and sintered in static and ambient air at 1000 °C for 24 h. Then, the obtained powders were reground and calcined in static and ambient air at 1000 °C for 24 h to produce the sample. All the products were furnace-cooled to room temperature and ground by hand. The powder XRD patterns were collected on a Rigaku TTR-III diffractometer equipped with a Cu Kα radiation source (λ = 1.5418 Å). The SEM images were obtained on the field emission scanning electron microscope (JEOL-2010 SEM) operated at 5 kV. The SEM specimens were prepared by depositing the catalyst powders on conductive carbon adhesive tape. HRTEM images and SAED patterns were acquired using a transmission electron microscope (JEOL, JEM-ARM200F) with a spherical aberration corrector at an acceleration voltage of 200 kV. For TEM specimen preparation, the catalyst powders were firstly dispersed in ethanol by sonication and then dropped onto a carbon-coated molybdenum grid with micropipettes, followed by drying under ambient conditions. XPS were carried out on an ESCALAB 250 X -ray photoelectron spectrometer using an Al Kα X-ray source. For XPS analysis, the binding energies were calibrated by referencing the C 1 s peak to 284.8 eV and no background subtraction was performed. The nitrogen adsorption-desorption isotherms were recorded at 77 K according to the preset pressure procedure using a Micromeritics ASAP 2000 system. Prior to the measurements, the samples were outgassed at 300 °C under vacuum for 10 h. The total surface areas were deduced from the isotherm analysis over the range of the relative pressure from 0.05 to 0.20. ICP-AES was performed on a Perkin-Elmer Optima 3300DV ICP spectrometer. The temperature-dependent resistivities of pelleted samples were measured on a Quantum Design Physical Property Measurement System by using the four-probe method. O *K*-edge XAS were measured at the beamline BL12B of National Synchrotron Radiation Laboratory (NSRL, Hefei) in the total electron yield mode by collecting the sample drain current under a vacuum better than 10^−7^ Pa. The beam from the bending magnet was monochromatized by utilizing a varied line-spacing plane grating and refocused by a toroidal mirror. The spectra from 525 to 550 eV were scanned with an energy resolution of 0.2 eV. To eliminate the effect of different sample concentration and measurement conditions on the intensity of XAS peaks, all the spectra were normalized to the adsorption background at the energy ranges both below the absorption edge and at ∼550 eV as described in the literatures^27,28,31^. The XAS specimens were prepared by placing a small amount of catalyst powders on a conductive carbon tape, which was mounted on an aluminum holder attached to the main chamber manipulator.

### Electrochemical measurements

The electrochemical tests were performed with a three-electrode on the CHI660E electrochemical station. Saturated Hg/Hg_2_SO_4_ and platinum wires were used as the reference and the counter electrodes, respectively. The Hg/Hg_2_SO_4_ reference electrode was calibrated in H_2_-saturated 0.5 M H_2_SO_4_ solution with a Pt wire as working electrode. The measured potentials vs Hg/Hg_2_SO_4_ were converted to the values with reference to a reversible hydrogen electrode (RHE). In 0.5 M H_2_SO_4_, *E*_RHE_ = *E*_Hg/Hg2SO4_ + 0.652 V. To prepare the working electrode, the catalysts (18.5 mg), activated carbon (3.7 mg), and Nafion (5 wt%, 100 μL) were dispersed in 5 mL tetrahydrofuran (THF) with sonication for 30 min to generate a homogenous ink. Then 5 μL ink was drop-casted onto a glassy carbon electrode of 3 mm in diameter and dried naturally, yielding a catalyst loading of 0.25 mg cm^−2^. Linear sweeping voltammograms were obtained at a scan rate of 5 mV s^−1^. The potentials are *іR*-corrected to compensate for the effect of solution resistance, which were calculated by the following equation:

1 $$E_{iR - {\mathrm{corrected}}} = E - iR,$$

where *i* is the current, and *R* is the uncompensated ohmic electrolyte resistance (~8 Ω) measured via high frequency ac impedance in O_2_-saturated 0.5 M H_2_SO_4_ solution. EIS measurements were carried out at different potential values with the frequency ranging from 100 kHz to 100 mHz under an AC voltage of 5 mV. The impedance spectra were presented in the form of Nyquist plot and fitted using ZView software with a representative equivalent electrical circuit. ECSA was determined by measuring the capacitive current associated with double-layer charging from the scan-rate dependence of cyclic voltammetry (CV)^10,17^. The potential window of CV was set to be 1.21–1.31 V vs RHE and the scan rates were 10, 20, 40, 60, 80, and 100 mV s^−1^. The double-layer capacitance (C_dl_) was estimated by plotting the Δj = (j_+_ − j_−_)/2 at 1.26 V vs RHE against the scan rate. The ECSA was calculated by the following equation:

2 $${\mathrm{ECSA = }}\left( {{\mathrm{C}}_{{\mathrm{dl}}}{\mathrm{/C}}_{\mathrm{s}}} \right){\mathrm{/M,}}$$

where C_s_ and M represented the specific capacitance and the loading mass for the catalyst, respectively. A C_s_ value of 60 μF cm^−2^ was used as the common estimate for oxide surfaces^38,39^. To conduct the long-term chronopotentiometric measurements, the catalysts were deposited on carbon paper with a mass loading of 0.25 mg cm^−2^ and followed by a heat-treating in ambient air at 300 °C. To carry out XRD and XPS measurements after the durability test for the catalysts, we followed the approaches frequently reported in recent literatures^7,12,13^, where the catalysts were loaded onto carbon paper with a high loading of 1 mg cm^−2^ for the durability test. After washing with ethanol and sonication, the specimens of ~2 mg were collected to use.

### Computational methods

All the density functional theory (DFT) calculations were performed with the Vienna Ab-initio Simulation Package (VASP). The projector augmented wave (PAW) potentials and Perdew-Burke-Ernzerhof (PBE) exchange-correlation functional were adopted. All calculations were conducted using a plane-wave kinetic energy cutoff of 520 eV. The energy convergence criterion was set to be 10^−5^ eV, and the force on each ion was converged to less than 0.05 eV/Å. The relevant details, models, and references are given in the Supplementary Methods section.

## Supplementary information

Supplementary Information

## Data Availability

The data that support the findings of this study are available from the corresponding author on request.

## References

[1] Carmo M, Fritz DL, Mergel J, Stolten D (2013). A comprehensive review on PEM water electrolysis. Int. J. Hydrog. Energy.

[2] Montoya JH (2017). Materials for solar fuels and chemicals. Nat. Mater..

[3] Park S, Shao Y, Liu J, Wang Y (2012). Oxygen electrocatalysts for water electrolyzers and reversible fuel cells: status and perspective. Energy Environ. Sci..

[4] McCrory CCL (2015). Benchmarking hydrogen evolving reaction and oxygen evolving reaction electrocatalysts for solar water splitting devices. J. Am. Chem. Soc..

[5] Seh ZW (2017). Combining theory and experiment in electrocatalysis: insights into materials design. Science.

[6] Lee Y, Suntivich J, May KJ, Perry EE, Shao-Horn Y (2012). Synthesis and activities of rutile IrO2 and RuO2 nanoparticles for oxygen evolution in acid and alkaline solutions. J. Phys. Chem. Lett..

[7] Grimaud A (2017). Activation of surface oxygen sites on an iridium-based model catalyst for the oxygen evolution reaction. Nat. Energy.

[8] Diaz-Morales O (2016). Iridium-based double perovskites for efficient water oxidation in acid media. Nat. Commun..

[9] Seitz LC (2016). A highly active and stable IrOx/SrIrO3 catalyst for the oxygen evolution reaction. Science.

[10] Yang L (2018). Efficient oxygen evolution electrocatalysis in acid by a perovskite with face-sharing IrO6 octahedral dimers. Nat. Commun..

[11] Lebedev D (2017). Highly active and stable iridium pyrochlores for oxygen evolution reaction. Chem. Mater..

[12] Shih P-C, Kim J, Sun C-J, Yang H (2018). Single-phase pyrochlore Y2Ir2O7 electrocatalyst on the activity of oxygen evolution reaction. ACS Appl. Energy Mater..

[13] Kim J (2017). High-performance pyrochlore-type yttrium ruthenate electrocatalysts for oxygen evolution reaction in acidic media. J. Am. Chem. Soc..

[14] Ebbinghaus SG, Weidenkaff A, Cava RJ (2002). Structural investigations of ACu3Ru4O12 (A = Na, Ca, Sr, La, Nd) -A comparison between XRD-Rietveld and EXAFS results. J. Solid State Chem..

[15] Subramanian MA, Sleight AW (2002). ACu3Ti4O12 and ACu3Ru4O12 perovskites: high dielectric constants and valence degeneracy. Solid State Sci..

[16] Su J (2018). Assembling ultrasmall copper-doped ruthenium oxide nanocrystals into hollow porous polyhedra: highly robust electrocatalysts for oxygen evolution in acidic media. Adv. Mater..

[17] Shang CY (2019). Electron correlations engineer catalytic activity of pyrochlore iridates for acidic water oxidation. Adv. Mater..

[18] Rossmeisl J, Qu Z-W, Zhu H, Kroes G-J, Nørskov JK (2007). Electrolysis of water on oxide surfaces. J. Electroanal. Chem..

[19] Man IC (2011). Universality in oxygen evolution electrocatalysis on oxide surfaces. ChemCatChem.

[20] Montoya JH, Doyle AD, Nørskov JK, Vojvodic A (2018). Trends in adsorption of electrocatalytic water splitting intermediates on cubic ABO3 oxides. Phys. Chem. Chem. Phys..

[21] Jacobs R, Hwang J, Shao-Horn Y, Morgan D (2019). Assessing correlations of perovskite catalytic performance with electronic structure descriptors. Chem. Mater..

[22] Dickens CF, Montoya JH, Kulkarni AR, Bajdich M, Nørskov JK (2019). An electronic structure descriptor for oxygen reactivity at metal and metal-oxide surfaces. Surf. Sci..

[23] Stamenkovic V (2006). Changing the activity of electrocatalysts for oxygen reduction by tuning the surface electronic structure. Angew. Chem. Int. Ed..

[24] Ling C, Shi L, Ouyang Y, Zeng XC, Wang J (2017). Nanosheet supported single-metal atom bifunctional catalyst for overall water splitting. Nano Lett..

[25] Petrykin V (2011). Zn-doped RuO2 electrocatalyts for selective oxygen evolution: relationship between local structure and electrocatalytic behavior in chloride containing media. Chem. Mater..

[26] Bolzan AA, Fong C, Kennedy BJ, Howard CJ (1997). Structural studies of rutile-type metal dioxides. Acta Cryst. B.

[27] Grimaud A (2013). Double perovskites as a family of highly active catalysts for oxygen evolution in alkaline solution. Nat. Commun..

[28] Suntivich J (2014). Estimating hybridization of transition metal and oxygen states in perovskites from O K-edge X-ray absorption spectroscopy. J. Phys. Chem. C..

[29] Mizumaki M (2013). Oxygen hole state in A-site ordered perovskite ACu3Ru4O12 (A = Na, Ca, and La) probed by resonant X-ray emission spectroscopy. J. Phys. Soc. Jpn..

[30] Xin Y, Zhou HD, Cheng JG, Zhou JS, Goodenough JB (2013). Study of atomic structure and electronic structure of an AA'3B4O12 double-perovskite CaCu3Ir4O12 using STEM imaging and EELS techniques. Ultramicroscopy.

[31] Crocombette JP, Pollak M, Jollet F, Thromat N, Gautier-Soyer M (1995). X-ray-absorption spectroscopy at the Fe L2,3 threshold in iron oxides. Phys. Rev. B.

[32] Nørskov JK (2004). Origin of the overpotential for oxygen reduction at a fuel-cell cathode. J. Phys. Chem. B..

[33] Rossmeisl J (2005). Electrolysis of water on (oxidized) metal surfaces. Chem. Phys..

[34] Xu Z, Rossmeisl J, Kitchin JR (2015). A linear response DFT + U study of trends in the oxygen evolution activity of transition metal rutile dioxides. J. Phys. Chem. C..

[35] Yang K (2018). Ultrasmall Ru/Cu‐doped RuO2 complex embedded in amorphous carbon skeleton as highly active bifunctional electrocatalysts for overall water splitting. Small.

[36] Spöri C, Kwan JTH, Bonakdarpour A, Wilkinson DP, Strasser P (2017). The stability challenges of oxygen evolving catalysts: towards a common fundamental understanding and mitigation of catalyst degradation. Angew. Chem. Int. Ed..

[37] Grimaud A (2017). Activating lattice oxygen redox reactions in metal oxides to catalyse oxygen evolution. Nat. Chem..

[38] Bockris JO, Otagawa T (1984). The electrocatalysis of oxygen evolution on perovskites. J. Electrochem. Soc..

[39] Da Silva LM, De Faria LA, Boodts JFC (2001). Determination of the morphology factor of oxide layers. Electrochim. Acta.

