# Supplementary material for: Quadruple perovskite ruthenate as a highly efficient catalyst for acidic water oxidation
Source: Nat Commun. 2019 Aug 23;10:3809. doi: 10.1038/s41467-019-11789-3 (PMC6707249; doi:10.1038/s41467-019-11789-3)
Supplement: Supplementary file 1 — Supplementary Information [file 41467_2019_11789_MOESM1_ESM.pdf]

Supplementary Information

# Quadruple perovskite ruthenate as a highly efficient catalyst for acidic water oxidation

Xianbing Miao, Lifu Zhang, Liang Wu, Zhenpeng Hu, Lei Shi, Shiming Zhou

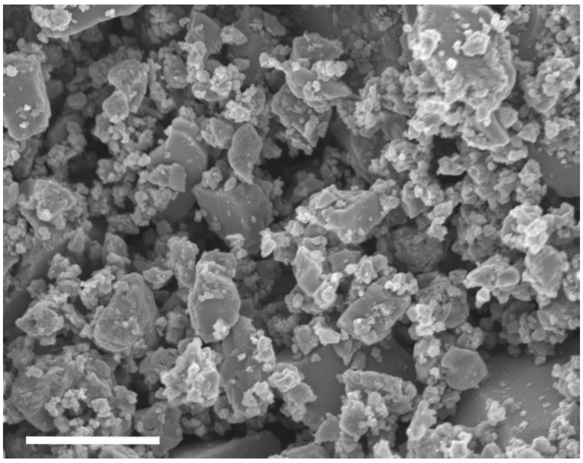

**Supplementary Figure 1 | SEM image of $CaCu_3Ru_4O_{12}$.** The scale bar is 1 μm.

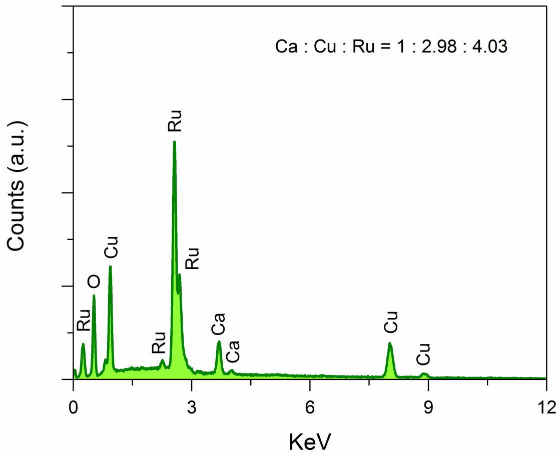


**Supplementary Figure 2 | EDS spectrum of $CaCu_3Ru_4O_{12}$.**

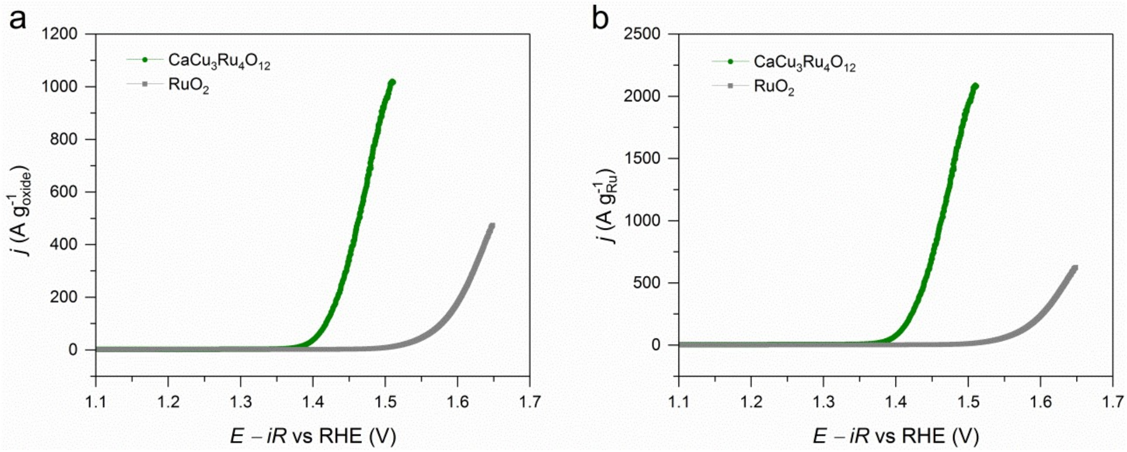


**Supplementary Figure 3 | Mass activities for $CaCu_3Ru_4O_{12}$ and the commercial $RuO_2$.**

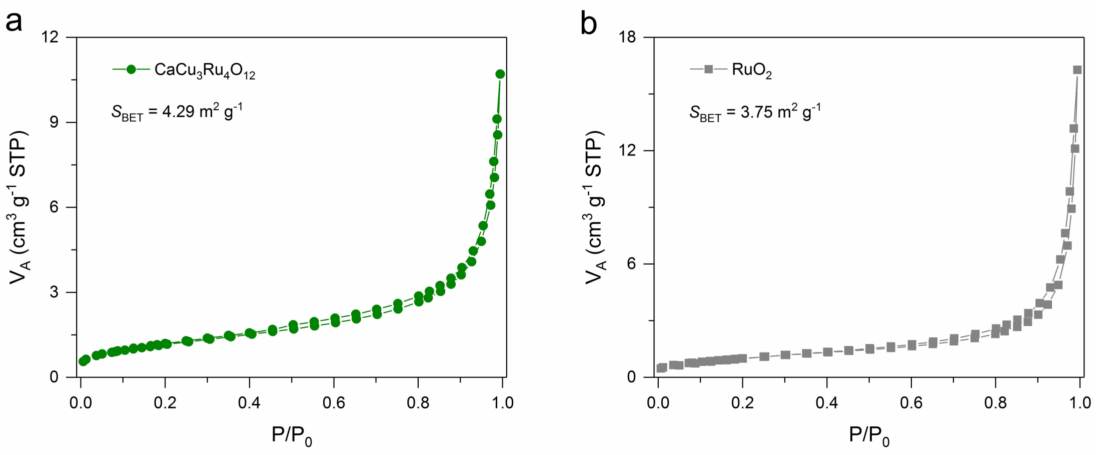


**Supplementary Figure 4 | Nitrogen adsorption-desorption isotherm curves.** (**a**) $CaCu_3Ru_4O_{12}$. (**b**) The commercial $RuO_2$.

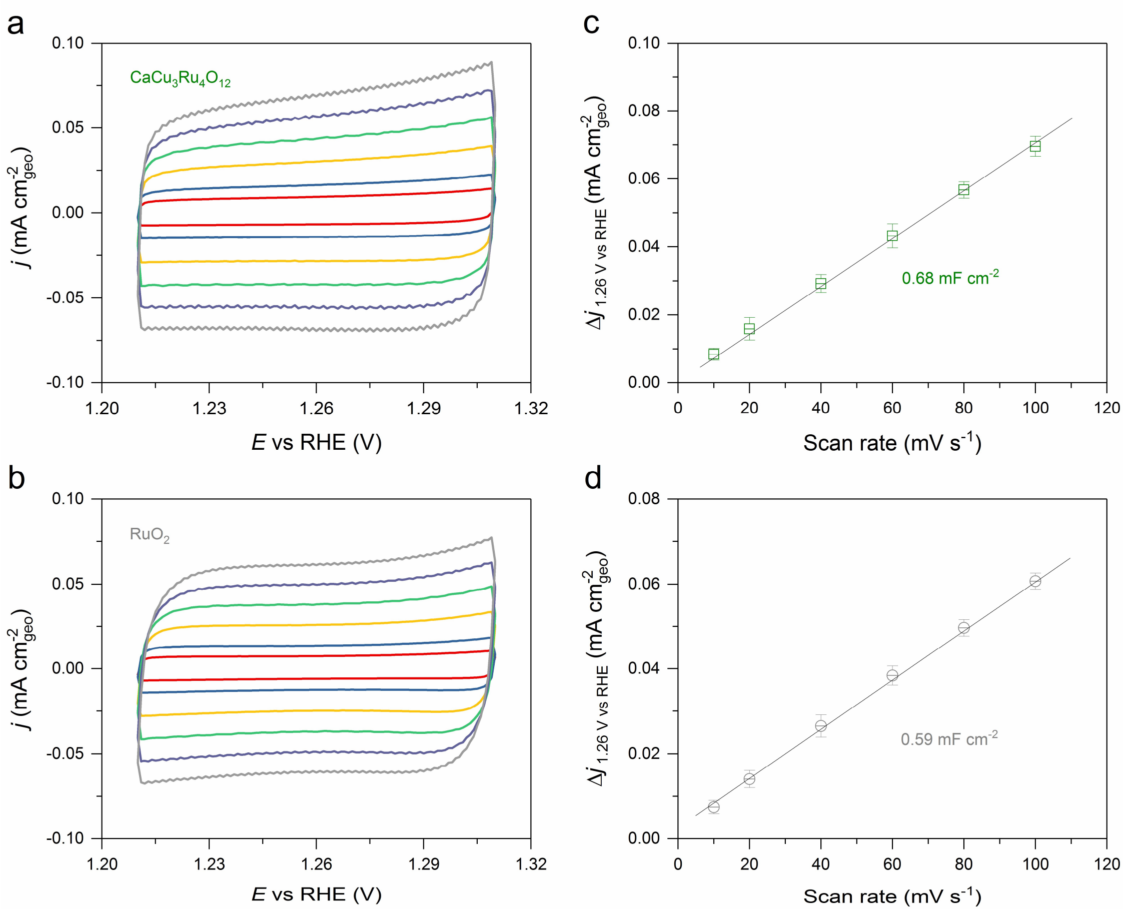


**Supplementary Figure 5 | ECSA analyses of $CaCu_3Ru_4O_{12}$ and the commercial $RuO_2$.** (**a**) and (**b**) CVs at different scan rates of in a potential window where no Faradaic processes occur (1.21-1.31 V vs RHE) for $CaCu_3Ru_4O_{12}$ and $RuO_2$, respectively. (**c**) and (**d**) Charging current density differences ($\Delta j = (j_+ - j_-)/2$) at 1.26 V vs RHE plotted against scan rates for $CaCu_3Ru_4O_{12}$ and $RuO_2$, respectively. The linear slope is equivalent to the double-layer capacitance ($C_{dl}$).

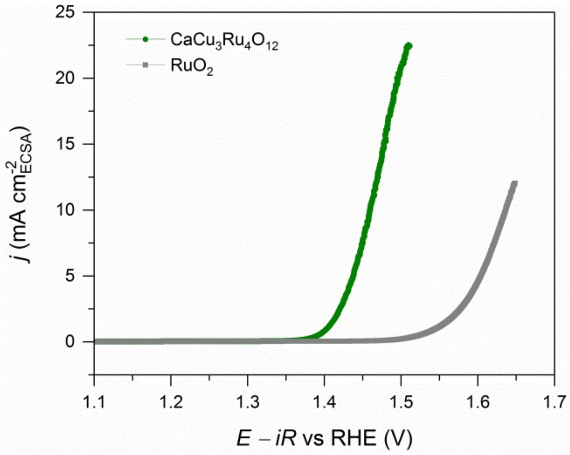


**Supplementary Figure 6 | Polarization curves for $CaCu_3Ru_4O_{12}$ and the commercial $RuO_2$ normalized by ECSA.**

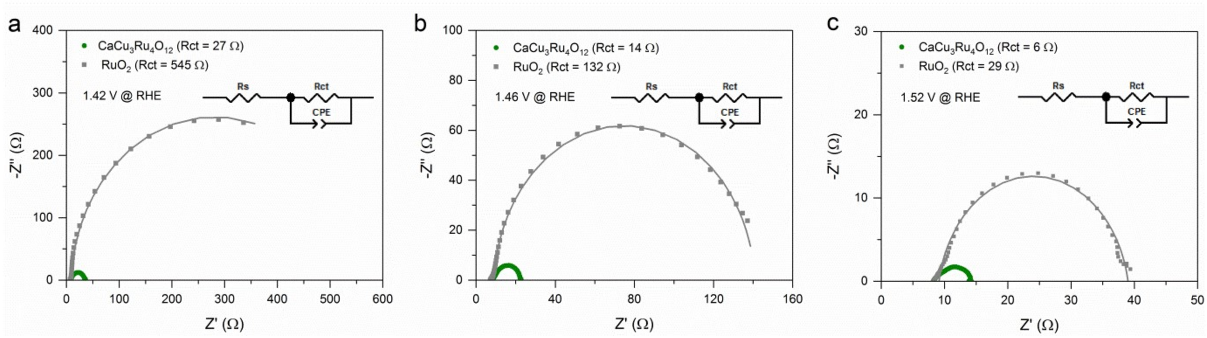


**Supplementary Figure 7 | Nyquist plots of $CaCu_3Ru_4O_{12}$ and the commercial $RuO_2$ at different voltages.** All impedance data were well fitted with the equivalent electrical circuit model (solid curves), which is composed of three components: an electrolyte resistance (Rs), a charge transfer resistance (Rct), and a constant-phase element (CPE).

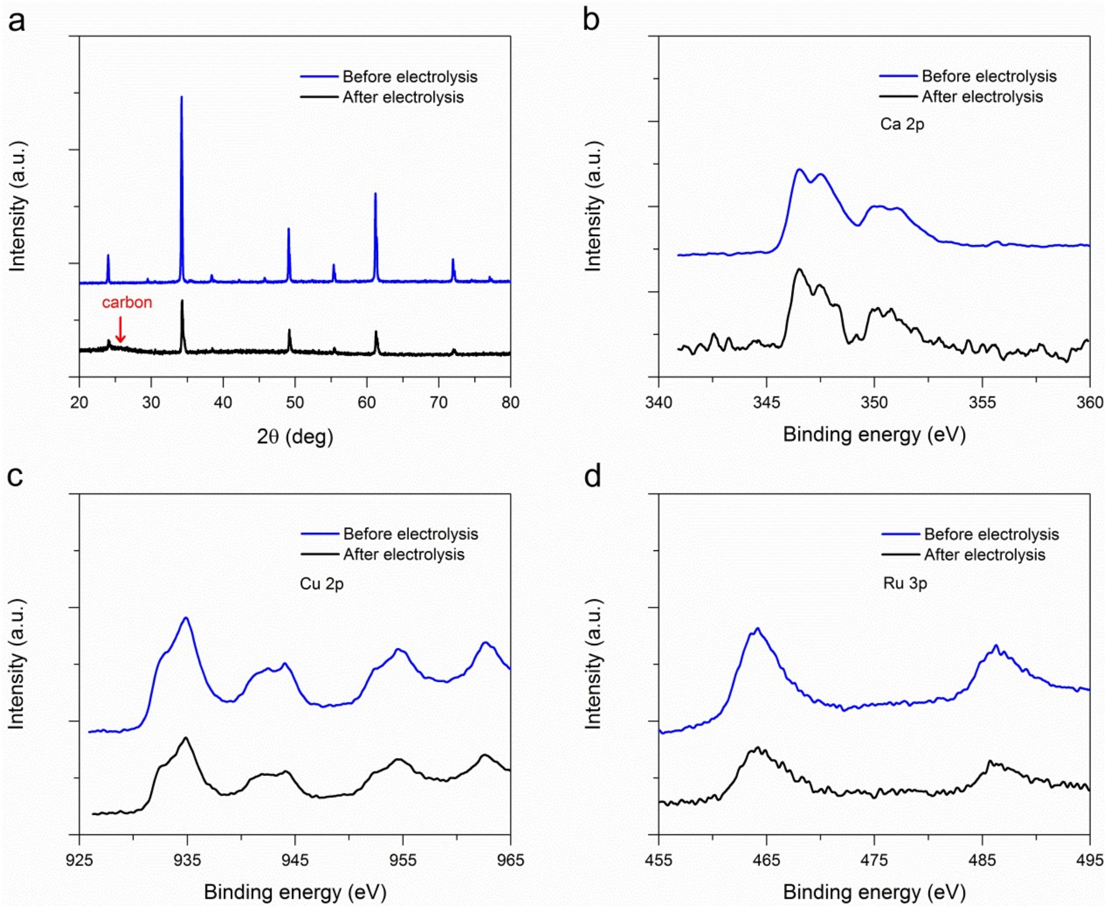


**Supplementary Figure 8 | XRD and XPS characterizations of $CaCu_3Ru_4O_{12}$ before and after the OER testing.** (**a**) XRD patterns. (**b**) Ca 2p, (**c**) Cu 2p, and (**d**) Ru 3p XPS spectra.

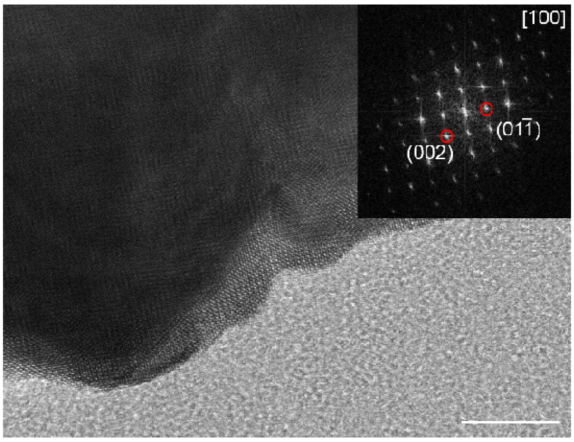


**Supplementary Figure 9 | HRTEM image for $CaCu_3Ru_4O_{12}$ after the durability test.** The inset is the corresponding fast Fourier transform (FFT).The scale bar is 10 nm.

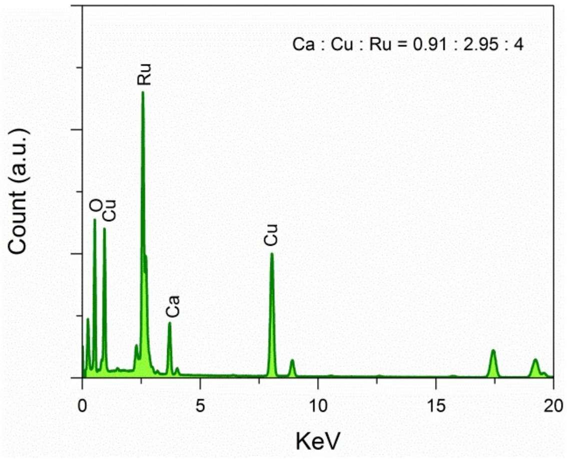


**Supplementary Figure 10 | TEM-EDS spectrum of $CaCu_3Ru_4O_{12}$ after the durability test.**

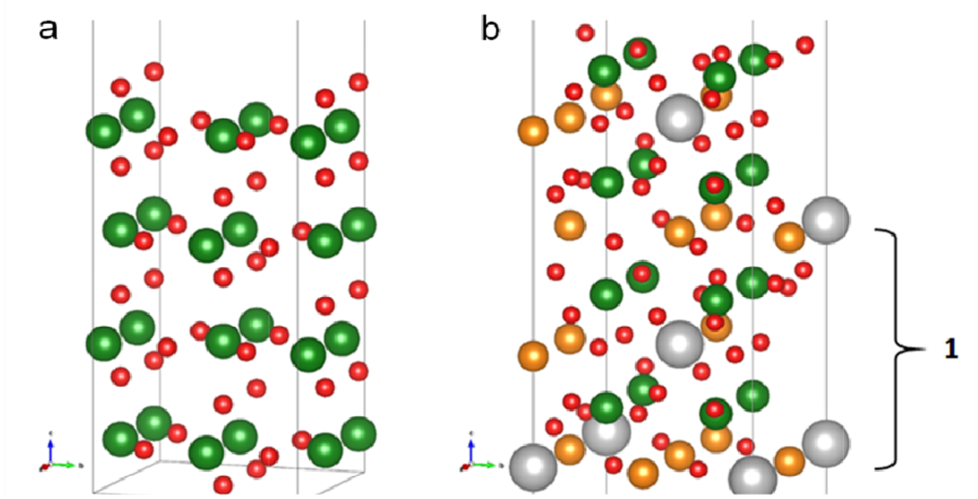


**Supplementary Figure 11 | Slab models for both catalysts.** (**a**) $RuO_2$ (110) and (**b**) $CaCu_3Ru_4O_{12}$ (001). Color code: Ca (gray), Cu (bronze), Ru (green), and O (red).

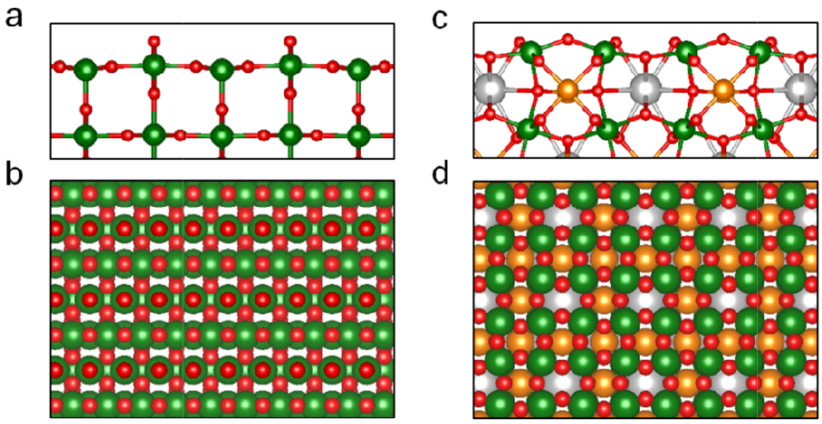


**Supplementary Figure 12 | Surface views for $RuO_2$ and $CaCu_3Ru_4O_{12}$.** (**a**) Side and (**b**) top views of $RuO_2$ (110). (**c**) Side and (**d**) top views of $CaCu_3Ru_4O_{12}$ (001). Color code: Ca (gray), Cu (bronze), Ru (green), and O (red).

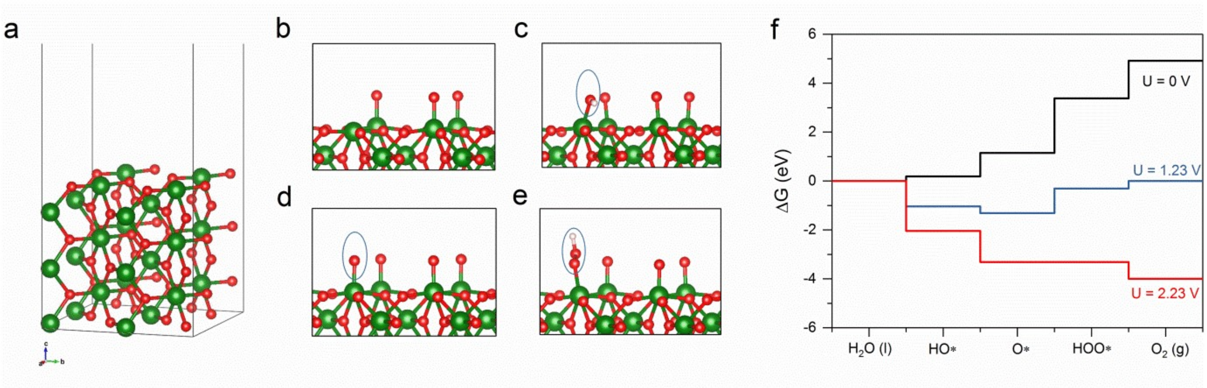


**Supplementary Figure 13 | DFT calculations.** (**a**) Slab models for $RuO_2$ (001). (**b-e**) Optimized structures of HO*, O*, and HOO* adsorptions on $RuO_2$ (001) surface, respectively. (**f**) Free energy diagram. Color code: Ru (green), and O (red).

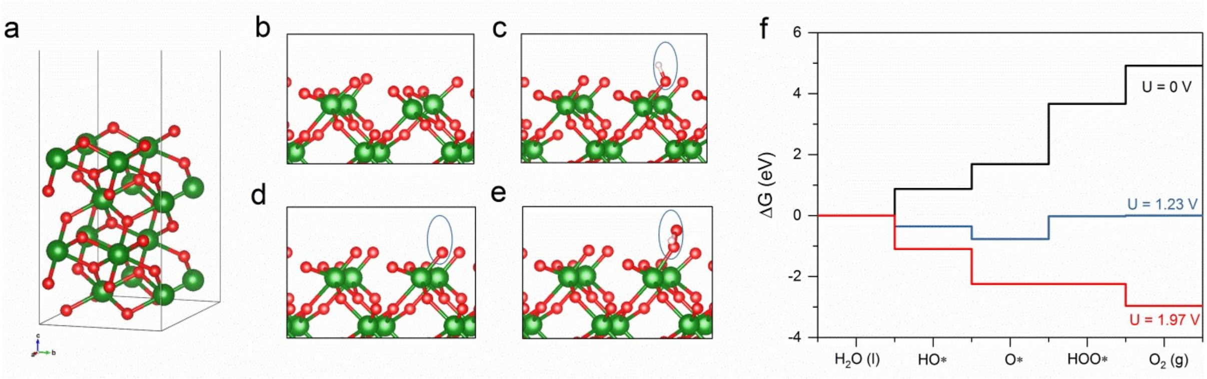


**Supplementary Figure 14 | DFT calculations.** (**a**) Slab models for $RuO_2$ (100). (**b-e**) Optimized structures of HO*, O*, and HOO* adsorptions on $RuO_2$ (100) surface, respectively. (**f**) Free energy diagram. Color code: Ru (green), and O (red).

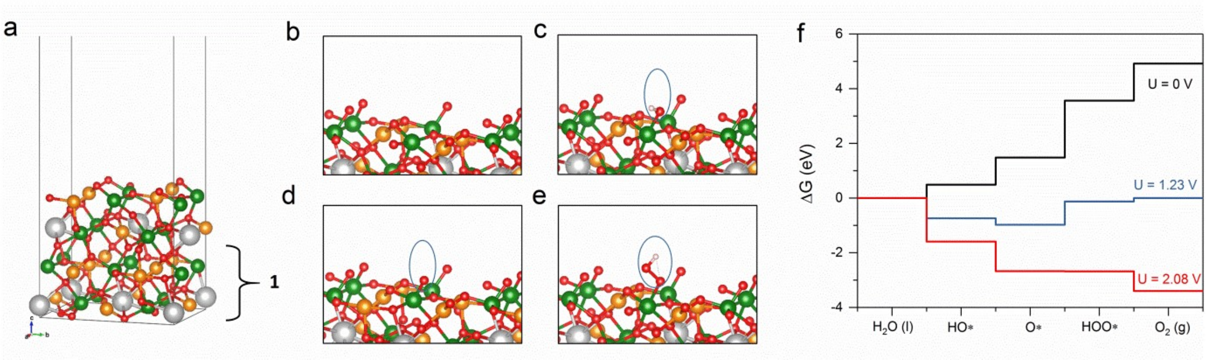


**Supplementary Figure 15 | DFT calculations.** (**a**) Slab models for $CaCu_3Ru_4O_{12}$ (110). (**b-e**) Optimized structures of HO*, O*, and HOO* adsorptions on Ru sites for the $CaCu_3Ru_4O_{12}$ (110) surface, respectively. (**f**) Free energy diagram. Color code: Ca (gray), Cu (bronze), Ru (green), and O (red).

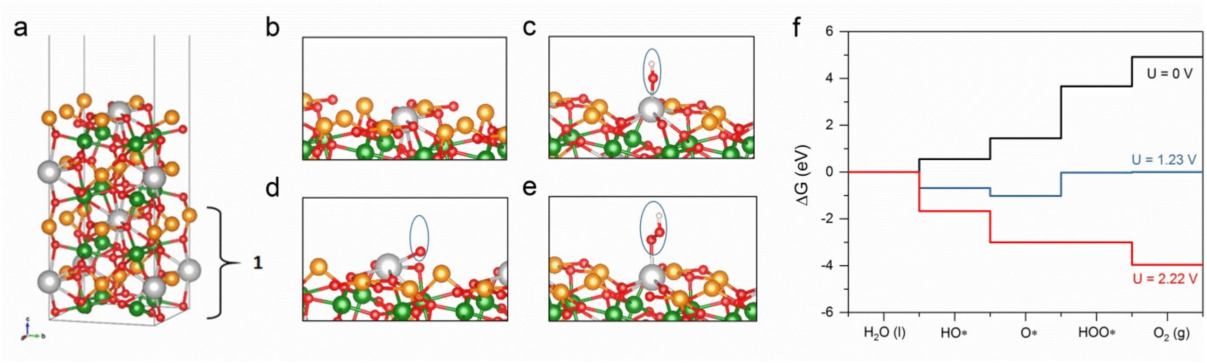


**Supplementary Figure 16 | DFT calculations.** (**a**) Slab models for $CaCu_3Ru_4O_{12}$ (001). (**b-e**) Optimized structures of HO*, O*, and HOO* adsorptions on Ca sites for $CaCu_3Ru_4O_{12}$ (001) surface, respectively. (**f**) Free energy diagram. Color code: Ca (gray), Cu (bronze), Ru (green), and O (red).

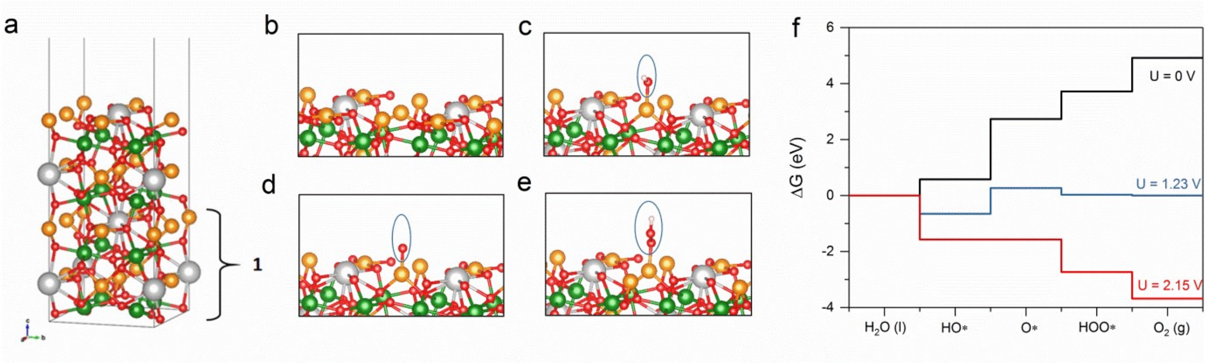


**Supplementary Figure 17 | DFT calculations.** (**a**) Slab models for $CaCu_3Ru_4O_{12}$ (001). (**b-e**) Optimized structures of HO*, O*, and HOO* adsorptions on Cu sites for $CaCu_3Ru_4O_{12}$ (001) surface, respectively. (**f**) Free energy diagram. Color code: Ca (gray), Cu (bronze), Ru (green), and O (red).

**Supplementary Table 1 | Refined structural parameters for $CaCu_3Ru_4O_{12}$.**

| | $CaCu_3Ru_4O_{12}$ |
|---|---|
| **Space group** | *Im-3* |
| **a (Å)** | 7.4206(3) |
| **V (Å$^3$)** | 408.622(5) |
| **$d_{Ru-O}$ (Å)** | 1.9808(7) |
| $R_{Bragg}$ | 4.87% |
| $R_p$ | 10.7% |

**a**: lattice parameter;

**V**: volume of unit cell;

$d_{Ru-O}$: Ru-O bond length;

$R_{Bragg}$ and $R_p$: goodness-of-fit parameters.

**Supplementary Table 2 | OER performances of $RuO_2$ reported in the literatures.**

| Catalyst | Substrate | Electrolyte | η (mV) @ *j* (mA $cm^{-2}$) | Tafel slop (mV $dec^{-1}$) | Reference |
|---|---|---|---|---|---|
| **$RuO_2$/C** | **GCE** | **0.5 M $H_2SO_4$** | **316@10** | **67** | **This work** |
| **$RuO_2$/C** | GCE | 0.1 M $HClO_4$ | 330@2.8 | 61 | 1 |
| **$RuO_2$ /acetylene black** | GCE | 0.1 M $HClO_4$ | 450@10 | ~ 64 | 2 |
| **$RuO_2$** | GCE | 0.1 M $HClO_4$ | 310@10 | - | 3 |
| **$RuO_2$** | GCE | 0.5 M $H_2SO_4$ | 297@10 | 64 | 4 |
| **$RuO_2$** | Au | 0.05 M $H_2SO_4$ | 370@10 | 73.7 | 5 |
| **$RuO_2$/C** | GCE | 0.1 M $HClO_4$ | 381@10 | 86 | 6 |
| **$RuO_2$** | GCE | 0.5 M $H_2SO_4$ | 298@10 | 64.47 | 7 |
| **$RuO_2$** | GCE | 0.5 M $H_2SO_4$ | 344@10 | 65 | 8 |
| **$RuO_2$** | GCE | 0.5 M $H_2SO_4$ | 289@10 | - | 9 |

**Supplementary Table 3 | Comparison of OER activities for the catalysts in acid media.**

| Catalysts | Electrolyte | Loading (mg cm$^{-2}$) | $\eta$ (mV) @ 10mA cm$^{-2}_{geo}$ | $j$ (mA cm$^{-2}_{oxide}$) @ 1.50 V | Reference |
|---|---|---|---|---|---|
| **$CaCu_3Ru_4O_{12}$** | **0.5 M $H_2SO_4$** | **0.250** | **171** | **22.1** | **This work** |
| **$Cr_{0.6}Ru_{0.4}O_2$** | 0.5 M $H_2SO_4$ | 0.279 | 178 | ~0.17@1.45V | 4 |
| **Cu doped $RuO_2$** | 0.5 M $H_2SO_4$ | 0.275 | 188 | ~0.18@1.45V | 7 |
| **carbon-supported hollow Pt/NiO/$RuO_2$** | 0.1 M $HClO_4$ | 0.014 | 239 | N/A | 6 |
| **6H-$SrIrO_3$** | 0.5 M $H_2SO_4$ | 0.90 | 248 | ~7.3 | 10 |
| **$RuO_2$nanowires** | 0.5 M $H_2SO_4$ | 0.171 | 250 | N/A | 8 |
| **Porous $Y_2[Ru_{1.6}Y_{0.4}]O_{7-\delta}$** | 0.1 M $HClO_4$ | 0.025 | 250 | ~2.2 | 11 |
| **N-WC nanoarray** | 0.5 M $H_2SO_4$ | 10.0 | 250 | N/A | 12 |
| **$IrO_x$/$SrIrO_3$** | 0.5 M $H_2SO_4$ | N/A | 270 | 10.0 | 13 |
| **$Co_3O_4$@C/CP** | 0.5 M $H_2SO_4$ | 5.0 | 270 | N/A | 14 |
| **$Pr_2Ir_2O_7$** | 0.1 M $HClO_4$ | 0.057 | 293 | 3.9 | 15 |
| **$RuO_2$** | 0.5 M $H_2SO_4$ | 0.250 | 316 | ~0.23 | This work |
| **$IrNiO_x$** | 0.05 M $H_2SO_4$ | 0.051 | 320 | N/A | 16 |
| **$Y_2Ru_2O_{7-\delta}$** | 0.1 M $HClO_4$ | 0.025 | 323 | ~1.5 | 1 |
| **$SrCo_{0.9}Ir_{0.1}O_{3-\delta}$** | 0.1 M $HClO_4$ | 0.255 | ~340 | ~3.0 | 17 |
| **$La_2LiIrO_6$** | 0.1 M $H_2SO_4$ | 0.250 | ~350 | ~0.70 | 18 |
| **Ba[Co-POM]** | 1 M $H_2SO_4$ | N/A | 361 | N/A | 19 |
| **$IrO_2$** | 0.1 M $HClO_4$ | 0.015 | 393 | ~0.08@1.53V | 20 |
| **$Ba_2NdIrO_6$** | 0.1 M $HClO_4$ | 0.015 | 480 | ~2.10@1.53V | 20 |
| **Co-$MoS_2$ nanosheets** | 0.5 M $H_2SO_4$ | 2.0 | 540 | N/A | 21 |
| **$Rh_2P$** | 0.5 M $H_2SO_4$ | 0.004 | 550 | N/A | 22 |
| **Ti-$MnO_2$** | 0.05 M $H_2SO_4$ | N/A | >670 | N/A | 23 |

**Supplementary Table 4 | ICP analysis of dissolved Ca, Cu, and Ru ions for $CaCu_3Ru_4O_{12}$ after the durability test.**

| Sample amount | 18 μg | 18 μg | 18 μg |
|---|---|---|---|
| Concentration of Ca ion (ppb) | 0.68 | 0.57 | 0.48 |
| Concentration of Cu ion (ppb) | 1.17 | 1.29 | 1.09 |
| Concentration of Ru ion (ppb) | 2.11 | 2.51 | 2.36 |
| Loss of mass (Ca) | 7.81% | 6.55% | 5.52% |
| Loss of mass (Cu) | 2.81% | 3.11% | 2.62% |
| Loss of mass (Ru) | 2.39% | 2.85% | 2. 68% |
| Average mass loss (Ca) | | 6.63% | |
| Average mass loss (Cu) | | 2.85% | |
| Average mass loss (Ru) | | 2.64% | |

## Supplementary Methods

**Computational details.** Density functional theory (DFT) calculations were carried out with the Vienna Ab-initio Simulation Package (VASP)[18–21]. The projector augmented wave (PAW) potentials[22] and Perdew-Burke-Ernzerhof (PBE) exchange-correlation functional[23] were adopted. All calculations were conducted using a plane wave kinetic energy cutoff of 520 eV. The energy converge criteria was set to be $10^{-5}$ eV, and the force was converged to less than 0.05 eV/Å on each ion. To simulate surfaces, we applied three-dimensional slab models in this work. The (110) surface of $RuO_2$ was represented by a p(2 × 1) slab with twelve atomic layers (Supplementary Fig. 11a). During the structural optimization, the bottom three layers were fixed, while the other nine atomic layers were allowed to relax. *K*-space was sampled using a 3×3×1 grid. The (001) surface of $RuO_2$ was represented by a p(1 × 1) slab with six atomic layers (Supplementary Fig. 13a). During the structural optimization, the bottom two layers were fixed, while the other four atomic layers were allowed to relax. *K*-spaces was sampled using a 3 × 3 × 1 grid. The (100) surface of $RuO_2$ was represented by a p(1 × 1) slab with twelve atomic layers (Supplementary Fig. 14a). During the structural optimization, the bottom six layers were fixed, while the other atomic layers were allowed to relax. *K*-spaces was sampled using a 2 × 3 × 1 grid. The (001) surface of $CaCu_3Ru_4O_{12}$ was represented by p(1 × 1) slab with two stoichiometric layers. During the structural optimization, the bottom layers (Supplementary Fig. 11b) were fixed, while the top layers were allowed to relax. *K*-spaces was sampled using a 3 × 3 × 1 grid. The (110) surface of $CaCu_3Ru_4O_{12}$ was represented by p(1 × 1) slab with two stoichiometric layers. During the structural optimization, the bottom layers (marked as 1 in Supplementary Fig. 15a) were fixed, while the top layers were allowed to relax. *K*-spaces was sampled using a 3 × 2 × 1 grid. The effect of metal ions Ca and Cu for (001) surface on OER activity have also been discussed. During the structural optimization, the bottom layers (marked as 1 in Supplementary Fig. 16a and 17a) were fixed, while the top layers were allowed to relax. *K*-spaces for both calculations were sampled using a 3 × 3 × 1 grid. The Ru 4d- and O 2p-band centers for $RuO_2$ and $CaCu_3Ru_4O_{12}$ were calculated by following the approach as described in the literatures[24–26].

**Gibbs free energies and theoretical overpotentials.** The mechanism of OER is assumed to proceed through four consecutive proton and electron transfer steps with HO*, O*, and HOO* intermediates, as shown below in acidic conditions.

$$H_2O\ (l) + * \rightarrow HO* + H^+ + e^- \quad (1)$$

$$HO* \rightarrow O* + H^+ + e^- \quad (2)$$

$$H_2O\ (l) + O* \rightarrow HOO* + H^+ + e^- \quad (3)$$

$$HOO* \rightarrow O_2\ (g) + * + H^+ + e^- \quad (4)$$

where * represents the surface active site. The free energy of the various intermediates at 298.15 K can be calculated by the following equation:

$$\Delta G = \Delta E + \Delta ZPE - T\Delta S - eU \quad (5)$$

where $\Delta E$ is the binding energy of adsorption species HO*, O*, and HOO*; $\Delta ZPE$, $\Delta S$, $U$ are the zero point energy changes, the entropy changes and the applied electrode voltage, respectively. The Gibbs free energy change of each OER step can then be obtained by calculating the binding energy of HO*, O*, and HOO* intermediates adsorbed on the catalyst surface. As a result, the pH independent theoretical overpotential, $\eta_{theory}$, is obtained as

$$\eta_{Theory} = \max\{\Delta G_{HO*}, \Delta G_{O*} - \Delta G_{HO*}, \Delta G_{HOO*} - \Delta G_{O*}, \Delta G_{O2(g)} - \Delta G_{HOO*}\}/e - 1.23\ V \quad (6)$$

## Supplementary References


1. Lin, Y. C. et al. Chromium-ruthenium oxide solid solution electrocatalyst for highly efficient oxygen evolution reaction in acidic media. *Nat. Commun*. **10**, 162 (2019).
2. Su, J. et al. Assembling ultrasmall copper-doped ruthenium oxide nanocrystals into hollow porous polyhedra: highly robust electrocatalysts for oxygen evolution in acidic media. *Adv. Mater.* **30**, 1801351 (2018).
3. Oh, A. et al. Topotactic transformations in an icosahedral nanocrystal to form efficient water-splitting catalysts. *Adv. Mater*. **31**, 1805546 (2019).
4. Yang, L.et al. Efficient oxygen evolution electrocatalysis in acid by a perovskite with face-sharing $IrO_6$ octahedral dimers. *Nat. Commun*. **9**, 5236 (2018).
5. Bhowmik, T., Kundu, M. K. & Barman, S. Growth of one-dimensional $RuO_2$ nanowires on g-carbon nitride: an active and stable bifunctional electrocatalyst for hydrogen and oxygen evolution reactions at all pH values. *ACS Appl. Mater. Interfaces* **8**, 28678-28688 (2016).
6. Kim, J., Shih, P.-C., Qin, Y., Al-Bardan, Z., Sun, C.-J. & Yang, H. A porous pyrochlore $Y_2[Ru_{1.6}Y_{0.4}]O_{7-\delta}$ electrocatalyst for enhanced performance towards the oxygen evolution reaction in acidic media. *Angew. Chem. Int. Ed*. **57**, 13877-13881 (2018).
7. Han, N. et al. Nitrogen-doped tungsten carbide nanoarray as an efficient bifunctional electrocatalyst for water splitting in acid. *Nat. Commun*. **9**, 924 (2018).
8. Seitz, L. C. et al. A highly active and stable $IrO_x/SrIrO_3$ catalyst for the oxygen evolution reaction. *Science* **353**, 1011-1014 (2016).
9. Yang, X. et al. Highly acid-durable carbon coated $Co_3O_4$ nano arrays as efficient oxygen evolution electrocatalysts. *Nano Energy* **25**, 42-50 (2016).
10. Shang, C. Y. et al. Electron correlations engineer catalytic activity of pyrochlore iridates for acidic water oxidation. *Adv. Mater*. **31**, 1805104 (2019).
11. Nong, H. N. et al. Oxide-supported $IrNiO_x$ core-shell particles as efficient, cost-effective, and stable catalysts for electrochemical water splitting. *Angew. Chem. Int. Ed*. **54**, 2975-2979 (2015).
12. Kim, J. et al. High-performance pyrochlore-type yttrium ruthenate electrocatalyst for oxygen evolution reaction in acidic media. *J. Am. Chem. Soc.* **139**, 12076-12083 (2017).

13. Chen, Y. B. et al. Exceptionally active iridium evolved from a pseudocubic perovskite for oxygen evolution in acid. *Nat. Commun*. **10**, 572 (2019).
14. Grimaud, A. et al. Activation of surface oxygen sites on an iridium-based model catalyst for the oxygen evolution reaction. *Nat. Energy* **2**, 16189 (2017).
15. Blasco-Ahicart, M., Soriano-López, J., Carbó, J. J., Poblet, J. M. & Galan-Mascaros, J. R. Polyoxometalate electrocatalysts based on earth-abundant metals for efficient water oxidation in acidic media. *Nat. Chem*. **10**, 24 (2018).
16. Diaz-Morales, O. et al. Iridium-based double perovskites for efficient water oxidation in acid media. *Nat. Commun*. **7**, 12363 (2016).
17. Xiong, Q. et al. One-step synthesis of cobalt-doped $MoS_2$ nanosheets as bifunctional electrocatalysts for overall water splitting under both acidic and alkaline conditions. *Chem. Commun*. **54**, 3859-3862 (2018).
18. Duan, H. et al. High-performance $Rh_2P$ electrocatalyst for efficient water splitting. *J. Am. Chem. Soc.* **139**, 5494-5502 (2017).
19. Frydendal, R., Paoli, E. A., Chorkendorff, I., Rossmeisl, J. & Stephens, I. E. L. Toward an active and stable catalyst for oxygen evolution in acidic media: Ti-stabilized $MnO_2$. *Adv. Energy Mater*. **5**, 1500991 (2015).
20. Kresse, G. & Furthmüller, J. Efficiency of *ab-initio* total energy calculations for metals and semiconductors using a plane-wave basis set. *Comput. Mater. Sci*. **6**, 15-50 (1996).
21. Kresse, G. & Furthmüller, J. Efficient iterative schemes for *ab initio* total-energy calculations using a plane-wave basis set. *Phys. Rev. B* **54**, 11169 (1996).
22. Kresse, G. & Hafner, J. *Ab initio* molecular dynamics for open-shell transition metals. *Phys. Rev. B* **47**, 558 (1993).
23. Kresse, G. & Hafner, J. *Ab initio* molecular-dynamics simulation of the liquid-metal-amorphous-semiconductor transition in germanium. *Phys. Rev. B* **49**, 14251 (1994).
24. Kresse, G. & Joubert, D. From ultrasoft pseudopotentials to the projector augmented-wave method. *Phys. Rev. B* **59**, 1758-1775 (1999).
25. Perdew, J. P., Burke, K. & Ernzerhof, M. Generalized gradient approximation made simple. *Phys. Rev. B* **77**, 3865 (1996).

26. Hammer, B. & Norskov, J. K. Why gold is the noblest of all the metals. *Nature* **376**, 238-240 (1995).
27. Hammer, B. & Nørskov, J. K. Electronic factors determining the reactivity of metal surfaces. *Surf. Sci*. **343**, 211-220 (1995).
28. Xin, H., Vojvodic, A., Voss, J., Nørskov, J. K. & Abild-Pedersen, F. Effects of d-band shape on the surface reactivity of transition-metal alloys. *Phys. Rev. B* **89**, 115114 (2014).
